# A Nationwide Survey of Bereaved Family Members' Perception of the Place Patients Spent Their Final Days: Is the Inpatient Hospice Like or Unlike a Home? Why?

**DOI:** 10.1089/pmr.2020.0058

**Published:** 2020-12-03

**Authors:** Hiroyuki Otani, Tatsuya Morita, Naoko Igarashi, Yasuo Shima, Mitsunori Miyashita

**Affiliations:** ^1^ Department of Palliative Care Team, and Palliative and Supportive Care, National Hospital Organization Kyushu Cancer Center, Fukuoka, Japan.; ^2^ Department of Palliative Care Team, and Palliative and Supportive Care, St. Mary's Hospital, Fukuoka, Japan.; ^3^ Department of Palliative and Supportive Care, Palliative Care Team, Seirei Mikatahara General Hospital, Hamamatsu, Shizuoka, Japan.; ^4^ Department of Palliative Nursing, Health Sciences, Tohoku University Graduate School of Medicine, Sendai, Miyagi, Japan.; ^5^ Department of Palliative Medicine, Tsukuba Medical Center Hospital, Tsukuba, Ibaraki, Japan.

**Keywords:** death, end-of-life environment, home, inpatient hospice

## Abstract

***Background:*** During end-of-life care, the place in which the patients spend time influences their quality of life.

***Objective:*** To clarify what it means to spend last days at home and in inpatient hospice.

***Design:*** This study was a part of a nationwide multicenter questionnaire survey of bereaved family members of cancer patients evaluating the quality of end-of-life care in Japan.

***Setting/Subjects:*** A nationwide questionnaire survey was conducted with 779 family members of cancer patients who had died at inpatient hospices. We asked participants about the perceived benefits of spending last days at home and inpatient hospice during the patient's last days.

***Measurements:*** A nationwide questionnaire.

***Results:*** Of participants, 37.6% (*n* = 185 [95% confidence interval, 33%–42%]) felt that the inpatient hospice was like a home. The family members who reported that the inpatient hospice felt like home significantly tended to report high satisfaction with the level of care (*p* < 0.01). Factors that the participants perceived as benefits of the inpatient hospice were: “If anything changes, as health care professionals are easily available, he/she can handle it” (88.1%), “he/she is reassured” (78.4%), and “he/she is safe” (72.7%). On the contrary, factors that they perceived as benefits of home were: “He/she can do what he/she wants to do without worrying about the eye of other people” (44.1%), “he/she can relax” (43.5%), and “he/she is free” (42.0%).

***Conclusions:*** Spending the last days of life in either an inpatient hospice or at home has specific benefits. The place a patient spends his/her end-of-life days should be based on patient and family values.

## Key Message

This article describes a nationwide survey of bereaved family members of cancer patients aimed at evaluating the quality of end-of-life care, especially the benefits of the place where the patients spend time, which influences their quality of life. The results reveal that the benefits of spending time at home were indicated by responses to the item: “He/she can do what he/she wants to do without worrying about the eye of other people” and “reminisce about the past and connect with his/her loved ones,” while the benefit of spending time at the inpatient hospice was: “Reassuring and safe as health care professionals are easily available.”

## Introduction

During end-of-life care, the place in which the patients spend time influences their quality of life.^1^ The concept of home has been explored over the past decade by different disciplines regarding what it means to spend time at home. The meaning of home has ontological and social significance and is regarded as “safe,” “secure,” “private,” “a place of reflection of the person's ideas and values,” “a place of emotional experience and locus,” and “a place of assets and support for work and leisure activities.”^2–4^ Especially at the end of life, it has been reported that home is closely related to the patient's security.^5^ In these reports, attempts are often made to create a more home-like environment in institutional settings (palliative care wards, nursing homes, etc.). Recent studies have reported that they view home-like environments as supporting their spiritual expression and social interaction and allowing privacy and compassionate activities by staff.^6,7^

On the other hand, the benefits of spending the end of life in the hospital reportedly included “feeling cared for and secure,” “receiving support to manage health,” “reassurance for family,” and “comfort,” and the benefits of spending time in the hospital were reported to extend beyond receiving treatment.^8,9^

It is useful to clarify the perceptions of the concepts of “inpatient hospice” and “home” in the end-of-life care setting in Japan to consider supportive measures in each setting. However, to date, few studies have explored this topic. This study aimed to focus on bereaved family members' perception of spending end-of-life moments at home and in an inpatient hospice.

## Methods

This study was a part of a nationwide survey (the Japan Hospice and Palliative Care Evaluation Study: J-HOPE 2016) of bereaved family members of cancer patients, aimed at evaluating the quality of end-of-life care across Japan.^10^ A multicenter questionnaire survey targeted the bereaved family members of cancer patients who had died at inpatient hospices. We mailed questionnaires to bereaved families in May 2016 and again in June 2016 to nonresponding families. Completion and return of the questionnaire were regarded as consent to participate in this study, and families who did not want to participate were asked to return the questionnaire with “no reply.” Ethical and scientific validity was confirmed by the institutional review boards of all the participating institutions. Each institutional review board/ethics committee determined that informed consent was not required, and the study was approved by the institutional review board/ethics review committees of the institutes to which the investigators belong.

### Setting and participants

The primary physicians identified potential participants with the following inclusion criteria: (1) bereaved family members of adult cancer patients (one family member was selected for each patient), (2) aged 20 years or older, (3) capable of completing a self-reported questionnaire, and (4) aware of the diagnosis of malignancy. Exclusion criteria were as follows: (1) inability to complete the questionnaire (dementia, cognitive failure, psychiatric illness, language difficulty, or visual loss), (2) patients' treatment-associated death or death in intensive care units, (3) unavailability of family member, (4) the patient having received palliative care services for less than three days, and (5) no serious psychological distress identified by the primary physician. As in previous studies,^11,12^ the final criterion was adopted on the assumption that primary physicians could identify families who might experience a serious psychological impact. However, given the aim of the present study, no formal criteria or psychiatric screening was applied. According to a previous research,^10^ families were surveyed 6–12 months after the patients' deaths.

### Measurements

The questionnaire was developed by the authors based on a literature review^1–9^ and discussion among the authors. The face validity of the questionnaire was confirmed through a pilot test with five bereaved family members and five physicians. The question (“To what extent did the inpatient hospice feel like home?”) was about how the participants perceived the home/inpatient hospice for bereaved family members; bereaved family members indicated the degree of agreement on a 4-point Likert-type scale of 1 (agree) to 4 (disagree). The respondents' level of satisfaction with care was rated on a 6-point Likert-type scale from 6 = strongly agree to 1 = disagree.

The questionnaire that related to what it means to spend final moments at home and in an inpatient hospice comprised 12 items evaluated in terms of the degree of agreement with the following statements on a 7-point Likert-type scale of 1 (strong benefits to home) −4 (unsure) −7 (strong benefits to inpatient hospice): “He/she can do what he/she wants to do without worrying about the eye of other people,” “he/she can relax,” “he/she is free,” “he/she can live his/her life the way he/she wants to,” “he/she can be in the place where his/her loved ones are,” “he/she can connect with his/her loved ones through daily activities,” “there is privacy and no one interferes,” “he/she can relive important events and memories of the past,” “he/she can reflect on his/her life,” “he/she is safe as health care professionals are easily available,” “he/she is reassured as health care professionals are easily available,” and “if anything changes, as health care professionals are easily available, he/she can handle it.” We asked the family members to report the following demographic data: patients' age, sex, and tumor sites; family members' age, sex, and relationship with the patient; and duration of stay in an inpatient hospice.

### Statistical analyses

Descriptive statistics were used to present the characteristics. For comparisons, respondents were classified into two groups: family members who reported that the inpatient hospice felt “a lot like home” or “a little like home” and all other family members. Comparisons were performed using Student *t*-test for the level of satisfaction that the respondents expressed with the care. A *p*-value of 0.050 was regarded as being significant. We divided participants into two categories: (1) “Benefits of home” as indicated by responses of “strong benefits of home” and “benefits of home” and (2) “benefits of an inpatient hospice” as indicated by responses of “strong benefits of an inpatient hospice” and “benefits of an inpatient hospice.” We then used explanatory factor analysis using the principal method with a promax rotation. We also calculated Cronbach's alpha coefficients. All analyses were performed using SPSS version 19.0.

## Results

Of the 779 questionnaires sent to the bereaved family members, 574 were returned (response rate 73.6%). Of the 574 respondents, 83 declined participation in the study. Thus, we analyzed a total of 491 responses (63% of the obtained data). Background characteristics are summarized in Table 1.

**Table 1.** Characteristics of Participants (*n* = 491)

Patients		
Age (years), mean (SD)	74.3	11.4
Sex		
Male	261	53.1
Female	229	46.6
Primary cancer site		
Lung	113	23
Esophagus and stomach	77	15.7
Colon and rectum	70	14.2
Pancreas	46	9.3
Gallbladder	30	6.1
Brain, head, and neck	28	5.7
Liver	24	4.9
Breast	23	4.7
Kidney and bladder	18	3.7
Uterus and ovary	17	3.5
Prostate	10	2
Blood/lymph nodes	10	2
Others	24	4.9
Hospitalization duration (number of days), mean (SD)	39.6	55
Family caregivers		
Age (years), mean (SD)	62.4	11.6
Sex		
Male	148	30.1
Female	333	67.8
Relationship with patient		
Spouse	210	42.8
Child	189	38.5
Sibling	32	6.5
Parent	11	2.2
Others	40	8.1
Time spent with patient during the final week		
Every day	323	65.7
4–6 days	68	13.8
1–3 days	64	13.0
None	24	4.8

Values are mean ± SD, or *n* (%).

Total percentages do not equal 100% because of missing values.

SD, standard deviation.

### Perception of home/inpatient hospice among bereaved family members

In response to the question “To what extent did the inpatient hospice feel like home?” 37.6% (*n* = 185 [95% confidence interval, 33%–42%]) family members reported that it felt a lot like home (12.6%, *n* = 62) and a little like home (25.0%, *n* = 123). The family members who reported that the inpatient hospice felt like home significantly tended to report high satisfaction with the level of care (*p* < 0.01).

### End-of-life environment and concepts of “home” and “inpatient hospice”

Factors that the participants perceived as “strong benefits” and “benefits” of the inpatient hospice were: “If anything changes, as health care professionals are easily available, he/she can handle it” (88.1%, *n* = 433), “he/she is reassured as health care professionals are easily available” (78.4%, *n* = 385), and “he/she is safe as health care professionals are easily available” (72.7%, *n* = 357). In addition, factors that they perceived as “strong benefits” and “benefits” of home were: “He/she can do what he/she wants to do without worrying about the eye of other people” (44.1%, *n* = 217), “he/she can relax” (43.5%, *n* = 214), and “he/she is free” (42.0%, *n* = 206), respectively (Table 2).

**Table 2.** The End-of-Life Location and the Concepts of “Home” and “Inpatient Hospice” (*n* = 491)

	Benefits of home		Benefits of inpatient hospice	
	n	%	n	%
He/she can do what he/she wants to do without worrying about the eyes of other people.	217	44.1	59	12
He/she can relax.	214	43.5	76	15.5
He/she is free.	206	42.0	64	13
He/she can live his/her life the way he/she wants to.	201	40.9	69	14.1
He/she can be in a place where his/her loved ones are.	178	36.3	71	14.5
He/she can connect with his/her loved ones through daily activities.	176	35.8	58	11.8
There is privacy and no one interferes.	163	34.2	97	19.8
He/she can relive important events and memories of the past.	158	32.2	51	10.4
He/she can reflect on his/her life.	141	28.7	51	10.4
He/she is safe.	26	5.3	357	72.7
He/she is reassured.	17	3.5	385	78.4
If anything changes, he/she can handle it.	5	1.0	433	88.1

The values are percentages of participants who reported agree or strongly agree.

### Factor analysis

In the exploratory factor analysis with 12 items, a subdomain comprising five items was identified as “He/she can do what he/she wants to do without worrying about the eye of other people” (Cronbach's alpha coefficient = 0.93); another subdomain comprising four items was identified as “Reminisce about the past and connect with his/her loved ones” (Cronbach's alpha coefficient = 0.90); and a third subdomain comprising three items was identified as “Safety and security as health care professionals are easily available” (Cronbach's alpha coefficient = 0.93) (Table 3).

**Table 3.** Factor Validity of the Concepts of “Home” and “Inpatient Hospice”: Three Core Domains (*n* = 491)

	Standardized regression coefficients			Communality
	F1	F2	F3	
He/she can do what he/she wants to do				
He/she can do what he/she wants to do without worrying about the eyes of other people.	**0.923**	0.632	0.231	0.950
He/she can live his/her life the way he/she wants to.	**0.891**	0.607	0.253	0.916
He/she is free.	**0.892**	0.631	0.257	0.885
He/she can relax.	**0.874**	0.649	0.268	0.823
There is privacy and no one interferes.	**0.673**	0.6	0.264	0.487
Reminisce about the past and connect with his/her loved ones				
He/she can connect with his/her loved ones through daily activities.	0.603	**0.919**	0.231	0.981
He/she can be in a place where his/her loved ones are.	0.589	**0.846**	0.262	0.848
He/she can reflect on his/her life.	0.642	**0.777**	0.243	0.642
He/she can relive important events and memories of the past.	0.658	**0.771**	0.218	0.613
Safety and security				
He/she is reassured.	0.255	0.244	**0.973**	0.983
If anything changes, he/she can handle it.	0.134	0.155	**0.725**	0.749
He/she is safe.	0.338	0.307	**0.763**	0.723

Boldfaced numbers indicate attributes belonging to each domain.

## Discussion

This is the first large-scale study to clarify family perceptions of what it means for patients to spend the final days at home and in inpatient hospice**s**. Of family members surveyed, 37.6% felt that the inpatient hospice was like a home. For the family members who were with the patients during their end of life, the benefits of spending time at home were indicated by responses to the item: “He/she can do what he/she wants to do without worrying about the eye of other people,” and “reminisce about the past and connect with his/her loved ones,” while the benefit of spending time at the inpatient hospice was that it was “reassuring and safe as health care professionals are easily available.” Multiple studies have revealed that the key environmental factors shown to affect end-of-life care were those that improved (1) social interaction, (2) positive distractions, (3) privacy, (4) personalization and creation of a home-like environment, and (5) the ambient environment.^13^ The present study confirms that the benefits of spending time at home/inpatient hospice, including these factors, should be considered when caring for patients and family members in each place to improve the physical, psychological, social, and spiritual needs at the end of life.

In order for patients and their families to feel more at home in the inpatient hospice, it is necessary to consider ways to enhance the sense of freedom, privacy, and attachment to the past, which are elements of the meaning of spending final days at home; for example, health professionals knocking on a door to enter a room as if you were visiting a private home and finding ways to connect with the past such as bringing an album from home. It was also suggested that in order for patients and their families to perceive the benefits of an inpatient hospice at home, it is necessary to consider ways to enhance safety and security, which are elements of what it means to spend the end-of-life days in an inpatient hospice, that is, having a system in which they can consult a health care professional at any time in case of any change in their condition and obtain support to manage their health at home so that they can feel secure.

A limitation of this study is that only family members of patients who spent their final days in the “inpatient hospice” were asked what it meant to spend the end-of-life days at home and in the inpatient hospice. Families of patients who spent their final days at home may have a different kind of meaning. In the future, further research with family members of patients who spent their final days at home is necessary. Furthermore, this study was conducted in Japan; the results are likely to have been influenced by factors relating to the Japanese culture and therefore may not be applicable to other countries. However, similar results were observed in previous studies.^5,8,13^

In conclusion, during end-of-life care, the place in which patients spend their time impacts their quality of life, and it became clear that spending the last days of one's life in an inpatient hospice or home has its own meaning. The place a patient spends his/her end-of-life days should be based on patient and family values.

## Authors' Contributions

Conception/design: H.O.; provision of study material or patients: T.M., N.I., Y.S., and M.M.; collection and/or assembly of data: N.I. and M.M.; data analysis and interpretation: H.O. and T.M.; article writing: H.O.; final approval of article: H.O., T.M., N.I., Y.S., and M.M.

## Abbreviation Used

SD: standard deviation
